# Future climate projection across Tanzania under CMIP6 with high-resolution regional climate model

**DOI:** 10.1038/s41598-024-63495-w

**Published:** 2024-06-03

**Authors:** Dawido S. Magang, Moses A. Ojara, Lou Yunsheng, Philemon H. King’uza

**Affiliations:** 1 Jiangsu Key Laboratory of Agricultural Meteorology, Nanjing University of Information Science and Technology, Nanjing, 210044 Jiangsu China; 2 School of Ecology and Applied Meteorology, Nanjing University of Information Science and Technology, Nanjing, 210044 Jiangsu China; 3 Directorate of Training and Research, Uganda National Meteorological Authority, Plot 21, 28 Port Bell Rd, P.O.BOX 7025, Kampala, Uganda; 4 School of Atmospheric Science, Nanjing University of Information Science and Technology, 219 Ningliu Road, Nanjing, 210044 Jiangsu China

**Keywords:** Climate sciences, Environmental sciences

## Abstract

Climate change is one of the most pressing challenges faced by developing countries due to their lower adaptive capacity, with far-reaching impacts on agriculture. The mid-century period is widely regarded as a critical moment, during which adaptation is deemed essential to mitigating the associated impacts. This study presents future climate projections across Tanzania using the latest generation of global climate models (CMIP6) combined with a high-resolution regional climate model. The findings indicate that, the trends in temperature and precipitation in Tanzania from 1991 to 2020, minimum temperatures showed the highest variability with a trend of 0.3 °C, indicating significant fluctuations in minimum temperature over the decades. Maximum temperatures also showed high variability with a trend of 0.4 °C. There is a range of variability in precipitation per decade for different regions in Tanzania, with some regions experiencing significant decreases in precipitation of up to − 90.3 mm and − 127.6 mm. However, there were also regions that experienced increases in precipitation, although these increases were generally less than 4.8 mm over the decades. The projections of minimum and maximum temperatures from 2040 to 2071 under the Shared Socioeconomic Pathways (SSP) 2–4.5 and SSP 5–8.5 are projected to increase by 0.14 °C to 0.21 °C per decade, across different regions. The average projected precipitation changes per decade vary across regions. Some regions are projected to experience increases in precipitation. Other regions are projected to show decreases in precipitation within the range of − 0.6 mm to 15.5 mm and − 1.5 mm to 47.4 mm under SSP2–4.5 and SSP5–8.5 respectively. Overall, both scenarios show an increase in projected temperatures and precipitation for most regions in Tanzania, with some areas experiencing more significant increases compared to others. The changes in temperatures and precipitation are expected to have significant impacts on agriculture and water resources in Tanzania.

## Introduction

Climate change is a pressing global issue that affects societies, economies, and ecosystems around the world^1–5^. In sub-Saharan Africa, the impacts of climate change are particularly profound, posing challenges to sustainable development, food security, and water resources^6,7^. Tanzania, located in East Africa, is vulnerable to the multiple threats posed by a changing climate, including shifts in rainfall patterns, rising temperatures, and an increased frequency of extreme weather events^8–12^.

The latest generation of climate models, known as Couple Model Intercomparison Project Phase 6 (CMIP6), provides valuable insights into future climate projections and helps to improve our understanding of potential climate change impacts on regional scales^13^. These models simulate various aspects of the Earth’s climate system and provide crucial information for policymakers and stakeholders to develop effective adaptation strategies^14,15^. In this study, we aim to investigate future climate projections across Tanzania using high-resolution regional climate models driven by outputs from the CMIP6 models. By employing a high-resolution regional climate model tailored to the specific characteristics of the Tanzanian regions, we can enhance the accuracy and spatial resolution of our projections, providing valuable information for decision-makers at the local, regional, and national levels.

Downscale is a crucial process in climate modeling that involves obtaining higher-resolution information from Global Climate Models (GCMs) and applying it to smaller spatial scales^16^. GCMs are designed to simulate the behaviour of the Earth’s climate system on a global scale, but their course resolution limits their ability to capture local and regional climate accurately^17^. Downscaling helps to bridge this gap by providing more detailed information at smaller scales, which is essential for understanding local climate variability and its impacts on various sectors^18^. Regional Climate Models (RCMs) operate at higher resolutions than GCMs and provide more detailed simulations of regional climate processes. Downscaled data from GCMs can be used to initialise and drive RCMs, improving their performance and enhancing our understanding of regional climate dynamics^14,17^. There are two approaches to downscaling the output from GCM: Statistical downscaling and Dynamic downscaling^19^. Statistical downscaling methods are frequently employed to analyse regional or local-scale climate conditions using GCMs output, involving the establishment of statistical relationships between large-scale and local/regional climate variables based on historical observed data. Common techniques include multiple linear regression and principal component analysis to drive downscaled climate variables based on the assumption that past climate will remain valid in the future^20,21^. Despite this, statistical downscaling introduces its own source of error^22^, *with the quality of input data having an impact on the accuracy of downscaled outputs*. In contrast, dynamical downscaling is an approach used in climate modeling to obtain high-resolution climate information for a specific region by using outputs from global climate models (GCMs) as input for regional climate models (RCMs)^23–25^. Overall, dynamical downscaling of global climate models plays a vital role in bridging the gap between the broad-scale climate projections produced by GCMs and the specific localized information needed for regional planning and decision-making^26^.

This research builds upon previous studies on climate change in Tanzania^27–32^, and contributes to the growing body of knowledge on future climate scenarios in East Africa. The study by Ref.^28^ observed that future rainfall over the southwestern highlands, western regions, and eastern side of Lake Nyasa will decrease, while the northeastern highlands and coastal regions will experience a slight increase in rainfall. While, Ref.^27^ also, by using seventeen GCMs, revealed changes in temperature and observed that June to August temperatures across the country by the twenty-first century are going to be higher than in the late twentieth century by 2.2 °C to 2.6 °C. Furthermore, the ensemble range spans change in rainfall from − 4 to + 30% by the 2090s, and the ensemble median changes from + 7 to + 14%^29^. The annual increases in rainfall are similar across the whole country^29^, but seasonal patterns change is paradoxical. Increases in precipitation during the months of January to February (JF) affect a majority of the country, with a notable impact on the southernmost areas. Conversely, heightened rainfall during the months of March to May (MAM) and September or October to December (OND) is most significant in the northern regions of the country. During June to September (JJAS), rainfall intensifies in the northernmost parts, while diminishing in central and southern Tanzania. These observations indicate a general trend of increasing rainfall during the respective wet seasons in each geographical region.

The projected increased changes in minimum temperatures reported that for each 1 °C, rice yield is expected to decline by 10%^33^, and a rise of temperature of 2 °C could reduce maize yield by 13% and rice yield by 7%^34^. Rainfall decreases of 10% have been correlated with a 2% decrease in national gross domestic product (GDP)^35^. The fluctuations in climate patterns are anticipated to amplify with further increases in temperatures, resulting in diverse impacts on various geographical areas as a consequence of climate change. These include changes in wetness and dryness^36^.

By combining the strengths of CMIP6 models with high-resolution regional climate modelling techniques, we seek to provide a comprehensive assessment of potential climate change impacts in Tanzania, including changes in temperature, precipitation patterns, and trends within variability. Through this research, we can help policymakers in Tanzania develop and implement effective adaptation strategies to mitigate the impacts of climate change. By understanding how temperatures and precipitation patterns are likely to change in the coming years, policymakers can design infrastructure projects, agricultural practices, and water resource management plans that are resilient to future climatic conditions. The research findings can assist policymakers in conducting comprehensive risk assessments to identify vulnerable regions, populations, and sectors that are most susceptible to the impacts of climate change. This information is essential for prioritizing resource allocation, disaster preparedness, and early warning systems to enhance resilience against climate-related hazards. The research findings can inform policies related to natural resource management, such as forestry, fisheries, and wildlife conservation. By anticipating shifts in ecosystems, species distributions, and biodiversity patterns, policymakers can develop conservation plans and sustainable use strategies that account for changing climatic conditions in Tanzania.

The remaining sections of this study are structured as follows: “Data and methodology” section describes the study area, datasets, and methods used in the study. Meanwhile, “Results” section presents the main results and discussions for each section. Finally, the conclusions highlighted in “Conclusion” section.

## Data and methodology

### Study area description

**Figure 1:**
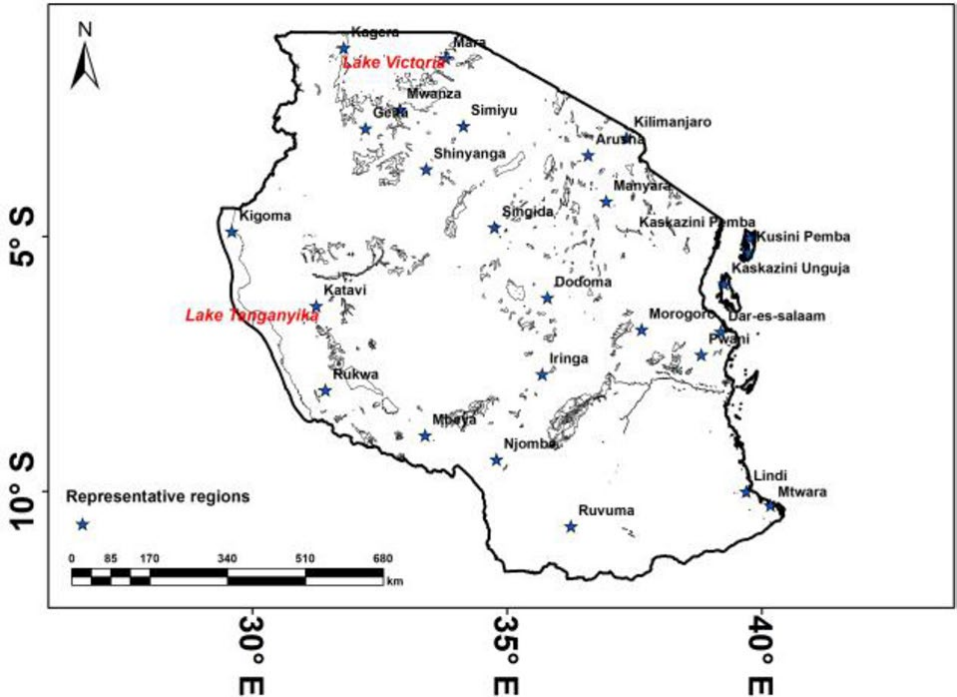
Study area. Blue stars denote twenty-four representative regions (Meteorological stations) across Tanzania.

Tanzania is a country located in the Eastern part of Africa (Fig. 1). It is bordered by Kenya and Uganda to the north, Rwanda, Burundi, and the Democratic Republic of the Congo to the west, Zambia, Malawi, and Mozambique to the south, and the Indian Ocean to the east. Its capital is Dodoma. Tanzania has an area of 947,300 square kilometres’^37^, 46% of which is arable land^38^. The current population is 61.7 million people^39^ and is expected to increase to 130 million people by 2050^10^. Tanzania is located by latitudes between 1° and 12° S and longitudes between 29° and 41° E. The main physical feature in Tanzania includes water bodies such as Lake Victoria, Tanganyika and Mountain Kilimanjaro etc.

Throughout the year, Tanzania experiences two distinct rainfall patterns: Bimodal and Unimodal. The short rainy *(Vuli)* in September or October to December (OND) and the long rains *(Msimu)* in January or February to May (MAM)^8^. The amount is usually 50 to 200 mm per month, but southwestern, central, southern and western parts of Tanzania received unimodal pattern rainfall. However, regions in the north, northern coast, Lake Victoria basin, north-eastern as well as the Island of Zanzibar used to receive two distinct seasonal rainfalls (bimodal)^8,28,40,41^.

During the wet season, the Intertropical Convergence Zone (ITCZ) moves southward, bringing moist air and rainfall to Tanzania. The seasonal migration of the ITCZ is sensitive to shifts in Indian Ocean Sea surface temperature and vary from year to year^42^, hence, the onset, duration and intensity of the rainfall vary considerably inter-annually. The monsoon wind is characterized by a reversal of the prevailing wind direction in the summer months, bringing moisture and rainfall to areas surrounding the Indian ocean, including Tanzania, which also causes seasonal variability^43^, as well as the areas outside the tropics (extra-tropical) characterized by variable weather conditions and frequent storms^43^. While the monsoon and extratropic regions are distinct meteorological phenomena, they can interact with each other. Land surface heterogeneity can cause local circulation systems by biophysical and topographic factors, which can have impacts on variability of rainfall and temperatures^44,45^.

The temperatures in Tanzania generally vary depending on the altitude of specific regions within the country^46^. Coastal areas exhibit distinct temperature patterns throughout the year. The dry season, which runs from June to October, experiences temperatures ranging from 27 to 29 °C, while the period from November to May typically experiences temperatures ranging from 28 to 32 °C. In the northern regions, the dry season (June to October) brings temperatures averaging between 20 and 30 °C, while the wet season (November to May) bring temperatures ranging from 15 to 27 °C^46^. In the southern highlands, including Iringa, Mbeya, and Njombe, the dry season temperatures range from 10 to 25 °C on average, with cooler temperatures compared to coastal and northern areas. The wet season in the southern highlands (November to May) is characterized by temperatures ranging from 20 to 22 °C on average. Western Tanzania, encompassing Kigoma and Katavi, experiences warm and dry weather during the dry season (June to October), with temperatures ranging from 20 to 30 °C. In contrast, the wet season (November to May) in these regions sees temperatures ranging from 22 to 32 °C on average^46,47^.

### Data

Several academic studies have utilized Regional Climate Models (RCMs) such as ICTP-RegCM4-3, MPI-CSC-REMO2009, and CCCma-CanRCM4 to analyze regional climate variations, impacts, and projections^48,49^. These RCMs are regarded as valuable tools for downscaling global climate model outputs to provide more detailed climate information at regional and local levels. The evaluation of the ability of these models to capture seasonal climate patterns in both present and future scenarios has been conducted through statistical significance tests as part of the Coupled Model Intercomparison Project Phase 6 (CMP6)^50^. The comparison of the simulated seasonal climate variables with actual data was carried out to ascertain the models’ proficiency in replicating current climate conditions^51^. These tests facilitated the assessment of the level of concordance between model forecasts and actual climate observations. The investigators identified regional inconsistencies in model accuracy, noting that specific models such as ICTP-RegCM4-3, MPI-CSC-REMO2009, and CCCma-CanRCM4 demonstrated superior performance in certain areas. Variations in replicating seasonal climate trends were evident in various geographical regions, underscoring the significance of evaluating models on a region-specific basis. The research also examined the efficacy of the models in predicting future climate alterations under varying scenarios. Through the evaluation of the models' accuracy in replicating current climate conditions and forecasting future patterns, scholars gauged the models' dependability in capturing extended climate fluctuations. The outcomes of the inquiry offer valuable insights into the efficacy and constraints of regional climate models in depicting seasonal climate trends^51–54^. A comprehensive understanding of the models’ performance is imperative for enhancing climate forecasts and guiding decision-making across diverse industries.

#### Model data

Climate modeling and simulations involve a wide range of disciplines, including atmospheric science, mathematics, computer science, and more to foster collaboration for model comparisons and impact assessments. Start by identifying individual or organizations with expertise in climate modeling and simulation, this can include the Coordinate Regional Climate Downscaling Experiment (CORDEX) that produces dynamical downscaled climate simulation for all continents and Climate Change Knowledge Portal (CCKP) under World Bank Group. In this study, dynamic downscaled climate simulations from a high resolution-regional climate model obtained from CMIP6 under CORDEX, accessed from https://www.nsc.liu.se/storage/esgf-datanode/ website, and CCKP, from https://climateknowledgeportal.worldbank.org/download-data website, were used to emerge the Tanzania climate change projection from 2040 to 2071: For monthly, and annual, minimum, maximum temperatures, and precipitation. CORDEX and CCKP collect all of their data at Earth System Grid Federation (ESGF), where the model operates over an equatorial domain with a quasi-uniform spatial resolution of approximately 50 × 50 km and model is set at longitude 0.44° and latitude 0.44° using a rotated pole system. Data accessibility of minimum, maximum temperatures, and precipitation for climate projections with two scenarios’: SSP 2–4.5 and SSP 5–8.5.

#### In-situ data

Data for Tanzania average monthly and annual precipitation and minimum, maximum temperatures was obtained from the Tanzania Meteorological Authority (TMA). The data collected from manual instruments, spans from 1950 to 2020.

### Methodology

#### Multi-model average

Climate models are tools for understanding and projecting future climates. However, they are subject to various uncertainties that can affect the accuracy and reliability of their projections^55^. Ensembles of models are often used to account for the range of possible outcomes^56^. In this study, the ensemble average or multi-model average methods have been utilized for the projection of the future climate pattern across Tanzania for the period from 2040 to 2071. This has been achieved by incorporating data from three different GCM-RCMs thereby bolstering confidence in the resulting predictions through the combination of models. The modal integrations selected to be used are depicted in Table 1.

**Table 1:** Details of CORDEX_RCMs and driving GCMs.

No.	RCM-name	Driving model	Ensemble	Experiment
1	ICTP-RegCM4-3	AFR-44_ECMWF_ERAINT	r1i1p1f1	SSP2–4.5 and SSP5–8.5
3	MPI-CSC-REMO2009	AFR-44_ECMWF_ERAINT	r1i1p1f1	SSP2–4.5 and SSP5–8.5
3	CCCma-CanRCM4	AFR-22_ECMWF_ERAINT	r1i1p1f1	SSP2–4.5 and SSP5–8.5

The Shared Socio-economic Pathways (SSPs) are set of scenarios that outline different possible future for human societies and their relationship with the natural world. They were adopted by the Intergovernmental Panel on Climate Change (IPCC) for its sixth Assessment Report (AR6) in 2021^57^. SSPs are designed to capture a range of social economic, and land use change that could occur over the next century^58^. Global warming of 2 °C would extremely likely be exceeded in the intermediate Greenhouse Gas (GHG); scenario SSP2–4.5 will reach its peak at around 2040 before starting to fall mid-century, but do reach net zero by 2100. While SSP5–8.5 is likely to occur under high emission throughout twenty-first century^59,60^. The SSP2–4.5 is a stabilization scenario where the radiative forcing level stabilises at 4.5 W/m^2^ before 2100 by the employment of a range of technologies and strategies for reducing greenhouse gas emissions^59^, and SSP5–8.5 at 8.5 W/m^2^ is consistent with a future with no policy changes to reduce emissions^61^. On account of processing of the available information, 24 representative regions (Meteorological stations) from Tanzania have selected (refer to Table S1).

Basically, spatial interpolation techniques are very important for creating continuous data from vector data, especially the point data^62^. Spatial interpolation is a mathematical technique that predicts based on input data^63^. This technique has been recommended from different studies for hydrological and meteorological applications^64,65^, which means detailed mathematical analysis of the techniques available in Ref.^66^. The Kriging interpolation method, which is based on spatially dependent variance, provides unbiased estimates of the target location in spacing using the known values of surrounding stations^67^. The Ordinary Kriging interpolation technique from ArcGIS is adopted in this study for patterns analysis from 50 to 5 km, which is based on data from about 24 regions.

#### The model’s skill rating

To determine the skill score of the model data, the Tanzania average annual observation data (in-situ) minimum, maximum temperatures, and precipitation were used. Pearson’s correlation coefficient is a measure of the strength and direction of the linear relationship between two variables, denoted by the symbol (r) (Eq. 1). In this case, the variables are model data (ICTP-RegCM4-3, MPI-CSC-REMO2009, and CCCma-CanRCM4) and average Tanzania observation data, both spanning from 1985 to 2014. To calculate the correlation coefficient, statistical software R was employed.

This coefficient ranges from − 1 to 1, where—1 indicates a perfect positive linear relationship, —0 indicates no linear relationship, and—− 1 indicates a perfect negative linear relationship^68^. The formula for computing the correlation (Pearson’s-r) is as follows:

1 $${\text{r}} = \frac{{\frac{1}{{{\text{n}} - 1}}\sum {\left( {{\text{X}} - \overline{{\text{X}}} } \right)} \left( {{\text{Y}} - \overline{{\text{Y}}} } \right)}}{{{\text{S}}_{{\text{X}}} {\text{S}}_{{\text{Y}}}}},$$

whereby, $\text{r}$ represent correlation between actual observational data and model forecast data, $\text{n}$ represents the sample size, $\text{X}$ for each historical observational data, $\overline{\text{X} }$ for mean. Likewise, $\text{Y}$ model forecast data, $\overline{\text{Y} }$ represent mean forecast data, and $\text{S}$ represent standard deviation for historical observation data and model forecast data.

To determine the significance of the correlation coefficient, Man-Kendal hypothesis test was employed^69^. The p-value associated with the correlation coefficient indicate whether the correlation is statistically significant or not. A low p-value (usually less than 0.05) suggests that the correlation is significant. Information regarding mathematical formulas can obtained from Ref.^70^.

One common skill score used in meteorology and forecasting is the correlation coefficient squared, also known as the coefficient of determination (R-squared) (Eq. 2). It represents the proportion of variance in the observed data that is predictable from the model data.

2 $$\text{R-squared}=\left({\text{r}}^{2}\right),$$

where by r represent correlation coefficient.

The skill score, represented by R-squared, ranges from 0 to 1. A score of 1 indicates that the model perfectly predicts the observation data, while a score of 0 indicates that the model does not explain any of the variability in the observation data.

## Results

### The model skill’s rating

The result concerning the model’s skill scores suggests that, MPI-CSC-REMO2009 model demonstrated strong performance in predicting minimum, maximum temperatures as well as precipitation under both SSP2–4.5 and SSP5–8.5 scenarios based on the coefficient of determination (R-squared) with historical average annual observation data from 1985 to 2014 (Table 2). The ICTP-RegCM4-3 model had moderate skill scores, while the CCCma-CanRCM4 model generally had the lowest skill scores. The skill scores varied across different scenarios and variables, indicating that model performance can differ based on the specific conditions being predicted. This indicates the model's robustness and reliability in capturing the trends and patterns of these climate variables within the specified scenarios over the given time period.

**Table 2:** Skill score results for the ICTP-RegCM4-3, MPI-CSC-REMO2009, and CCCma-CanRCM4 models.

Model names	Scenarios	Min. temperature (r)	Max. temperature (r)	Precipitation (r)	R-squared	p-value
ICTP-RegCM4-3	SSP2–4.5	0.55	0.58	0.48	0.86	0.9
	SSP5–8.5	0.56	0.50	0.47	0.78	0.3
MPI-CSC-REMO2009	SSP2–4.5	0.62	0.49	0.61	0.99	0.9
	SSP5–8.5	0.67	0.46	0.52	0.93	0.9
CCCma-CanRCM4	SSP2–4.5	0.50	0.68	0.51	0.97	0.9
	SSP5–8.5	0.58	0.49	0.30	0.66	0.3

The r-value for the correlation test between observed and simulated climate data, coefficient of the determination (R-squared) for the skill score, and the p-value for the significance test.

### Current climate (climatology)

#### Temperatures

**Figure 2:**
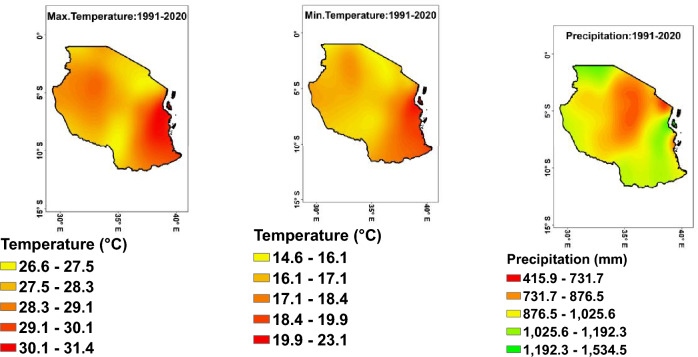
Observed current average climatology of maximum (left), minimum (middle) temperatures, and precipitation (right) from 1991 to 2020.

The current climatology of temperatures across Tanzania from 1991 to 2020 (Fig. 2) shows that, there is a range of temperatures across different regions. The lowest minimum temperature was recorded in Arusha (15.1 °C), followed by Mtwara (20.6 °C) and Lindi (20.3 °C). The highest minimum temperature was recorded in Zanzibar (22.7 °C). There is variability in minimum temperature across Tanzania, with some regions experiencing cooler temperatures compared to others (Fig. 2-middle). On the other hand, the maximum temperature from 1991 to 2020 (Fig. 2-left) reveals a range of temperatures recorded in different regions. The highest maximum temperature was observed in Pwani (31.3 °C) and Dar es Salaam (31.1 °C), while other regions with relatively high maximum temperatures include Zanzibar (31.0 °C), Lindi (30.3 °C), and Mtwara (30.3 °C). Conversely, cooler maximum temperatures were noted in regions such as Manyara (26.8 °C) and Njombe (26.7 °C). These findings highlight the variability in maximum temperatures across Tanzania, with coastal regions generally experiencing higher temperatures compared to inland areas.

#### Precipitation

The precipitation from 1991 to 2020 (Fig. 2-right) shows a wide range of precipitation levels across different regions. The highest precipitation levels were observed in Zanzibar at 1530 mm and the lowest in Dodoma at 635.2 mm. Dar es Salaam also had high precipitation levels above 1300 mm. Overall, there is variability in precipitation, with some regions experiencing higher levels of rainfall than others.

The regions with relatively consistent precipitation levels that may be suitable for agricultural purposes include Kagera, Mwanza, Mara, and Morogoro with 1327.6 mm, 1137.6 mm, 1140.6 mm, and 1155.2 mm of precipitation, respectively. These regions have shown consistent precipitation levels over the years, which can be beneficial for agricultural activities as they provide a reliable source of water for crops.

#### Seasonal temperatures and precipitation

From 1991 to 2020, the seasonal climatology across Tanzania showed distinct patterns in minimum, maximum temperatures as well as precipitation (Table 3). The months of October–November–December (OND) had the highest minimum temperature at 17.7 °C, while January–February (JF) had the highest at 18.7 °C. The highest maximum temperature was recorded in January–February at 28.9 °C, while the lowest were in June–July–August–September at 27.3 °C (JJAS). In terms of precipitation, the months of January–February (JF) and March–April–May (MAM) had the highest amount at 438.9 mm and 363.3 mm, respectively, while June–July–August–September had the lowest at 23.5 mm.

**Table 3:** Average seasonal minimum, maximum temperatures, and precipitation from 1991 to 2020.

	Jan–Feb	Mar–Apr–May	Jun–Jul–Aug–Sept	Oct–Nov–Dec
Average min. temperature (°C)	18.7	18.0	15.0	17.7
Average max. temperature (°C)	28.9	28.2	27.3	29.8
Precipitation (mm)	438.9	363.3	23.5	132.9

### Change and significance climatology

#### Temperatures and precipitation

**Figure 3:**
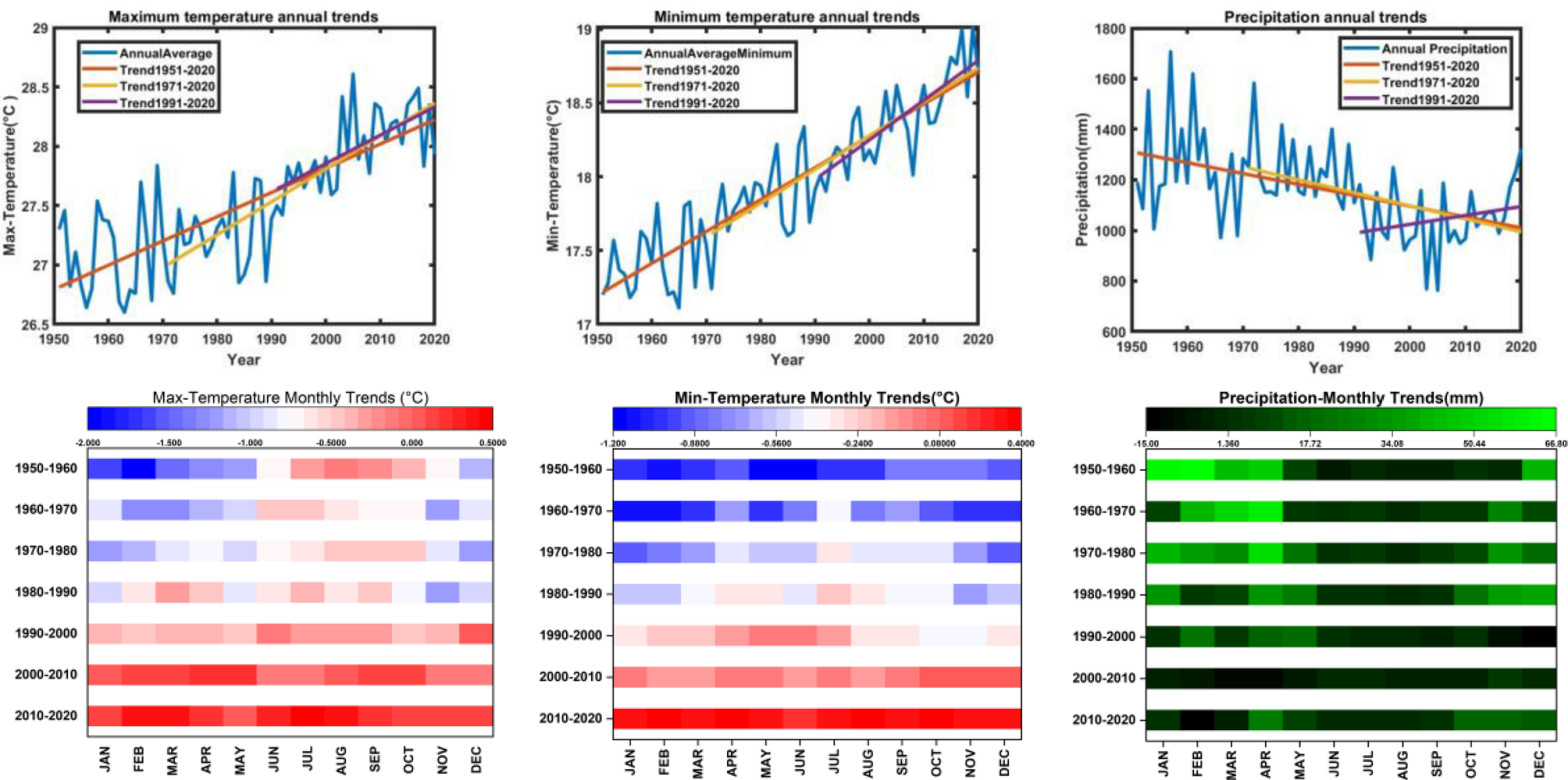
Annual trends (upper) of maximum (left), minimum (middle) temperatures, and precipitation (right) with significance trends and monthly trends per decade (lower).

From Fig. 3, top, the results show that minimum, maximum temperatures in Tanzania have been increasing significantly over the past few decades. The trend per decade for both minimum, maximum temperatures has been positive, with higher values in more recent decades. On the other hand, the trend for precipitation has been decreasing significantly over the same time period, with a notable increase in the trend for precipitation in the most recent decade (1991–2020) with 86% of the significance (Fig. 3, top-right). These findings suggest a changing climate in Tanzania with increasing temperatures and decreasing precipitation, which could have significant implications for the region’s ecosystems and water resources. Rising temperatures can alter the timing and length of growing seasons, affecting the planting and harvesting of crops^7,9^. This can lead to changes in agricultural calendars and require adjustments in farming practices. Decreasing precipitation can result in water scarcity, making irrigation more challenging for farmers. This can impact crop yields and the overall productivity of agriculture in the region.

From the monthly average temperatures trend data from 1951 to 2020 (Fig. 3, bottom) show a gradual increase in temperatures over the decades. From 1950 to 1960, temperatures were consistently below zero, with January being the coldest month. However, from 2000 to 2010, temperatures started to rise and by 2010–2020, there was a significant increase in minimum temperature across all months, with January and October experiencing the highest increase. This indicates a warming trend in the average minimum temperature over the period studied (Fig. 3, bottom-middle). Furthermore, a trend of increasing average maximum temperature from 1951 to 2020 (Fig. 3, bottom-left). The data indicates that there has been a gradual warming trend over the decades, with temperature generally becoming less negative or even positive in some months. The months of January, February, and March have shown the most significant increase in average maximum temperature over the years. From 1951–1960 to 2010–2020, the average maximum temperature trend shows a clear pattern of warming.

The warming trend in average maximum temperature aligns with the broader global climate change patterns observed over the past decades. Global climate change, driven largely by human activities such as the burning of fossil fuels and deforestation, has led to an overall increase in global temperatures^57,71^. This increase in temperatures is manifested in various ways, including a rising average maximum temperature, changes in precipitation patterns, and more frequent extreme weather events. The result showing a consistent warming trend in average maximum temperature from 1951 to 2020, is in line with the overall trend of global warming^1^. Scientists have documented that the Earth’s average temperature has been increasing steadily, with each decade since the 1970s being warmer than the previous one^1^. This warming trend is primarily attributed to the greenhouse gas emissions that trap heat in the Earth’s atmosphere, leading to the planet’s overall temperature rise. The impacts of this global warming trend are far-reaching and include rising sea levels, more intense heatwaves, changes in precipitation patterns, and shifts in ecosystems and biodiversity. These changes have significant implications for both the environment and human societies, affecting agriculture, water resources, public health, and economies around the world. Therefore, the warming trend in average maximum temperature that has been highlighted is a local manifestation of the broader global climate change patterns that are reshaping our planet. It underscores the urgent need for efforts to mitigate greenhouse gas emissions, adapt to the impacts of climate change, and work towards a sustainable future for generations to come^2,57,72^.

Furthermore, the average monthly precipitation trend from 1951 to 2020 (Fig. 3, bottom-right), shows variations in precipitation levels over the decades. From 1950–1960 to 2010–2020, there are fluctuations in the monthly precipitation amounts, with some months experiencing increases and others experiencing decreases. Overall, there is no consistent pattern in the trend, with some years showing higher levels of precipitation and others showing lower levels. There are several factors that can contribute to fluctuations in monthly and annual precipitation amounts over the years. Some of the key factors include: Climate change can have a significant impact on precipitation patterns. Changes in global temperatures can alter atmospheric circulation patterns, leading to shifts in precipitation distribution. Natural climate variability, such as El Niño and La Niña events, can influence precipitation patterns on a seasonal or even yearly basis. These phenomena can result in periods of increased or decreased precipitation. The geographic features of a region, such as mountains or plains, can affect precipitation patterns. Ocean circulation patterns, like the Indian Ocean Dipole (IOD), can influence atmospheric conditions and precipitation patterns. These patterns can lead to variations in rainfall amounts over time. Human activities, including the emission of greenhouse gases and aerosols, can contribute to changes in precipitation patterns. Climate change resulting from human-induced factors can alter atmospheric conditions and impact precipitation amounts^73–78^. By considering these various factors, it becomes possible to understand the complex interplay of natural and anthropogenic influences on precipitation fluctuations. Climate change can have profound effects on precipitation patterns, leading to shifts in the frequency, intensity, and distribution of rainfall across different regions. By understanding how climate change affects precipitation patterns, scientists and policymakers can better prepare for and adapt to the changes in precipitation that are expected to occur in the coming years.

### Trend per decade and significant change against natural variability

#### Temperatures

**Figure 4:**
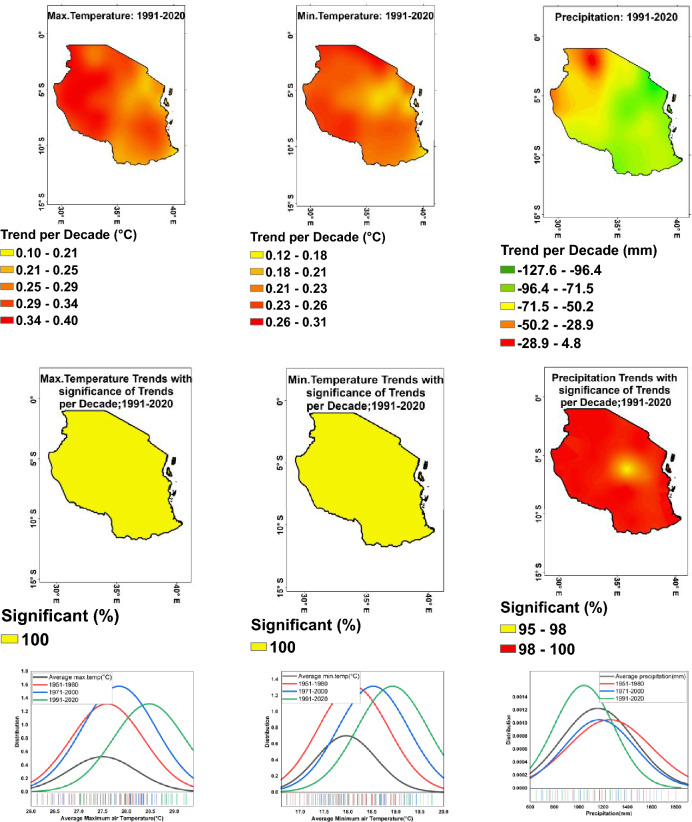
Average annual maximum (top-left), minimum (top-middle) temperatures, and precipitation (top-right) trend per decade from 1991 to 2020 corresponding with significance of trends per decade and change in distribution of average maximum (bottom-left), minimum (bottom-middle) temperatures, and precipitation (bottom-right) from 1951 to 2020.

The trends within variability from 1991 to 2020 for minimum and maximum temperatures per decade include: For minimum temperature (Fig. 4, top-middle), the regions of Geita, Kigoma, Rukwa, Mbeya, Kagera, Arusha, and Ruvuma showed the highest variability with a trend of 0.3 °C for each, indicating significant fluctuations in minimum temperature over the decades. Other regions, such as Tabora, Singida, Manyara, Katavi, and Mtwara, also displayed moderate variability in the minimum temperature trend. For maximum temperature (Fig. 4, top-left), the regions of Katavi and Tabora showed the highest variability with a trend of 0.4 °C each, indicating significant fluctuations in maximum temperature over the decades. The regions of Shinyanga, Geita, Kigoma, Rukwa, Mwanza, and Mara also displayed high variability in maximum temperature trends, with values ranging from 0.3 to 0.4 °C. Other regions, such as Singida, Dodoma, Morogoro, and Arusha, showed moderate variability in maximum temperature trends.

#### Precipitation

The results on trends within variability across Tanzania from 1991 to 2020 show that there is a range of variability in precipitation per decade for different regions (Fig. 4, top-right). Geita and Kigoma experienced some of the greatest decreases in precipitation over the decades, with decreases of − 90.3 mm and − 127.6 mm, respectively. Other regions, such as Manyara and Kilimanjaro, also saw significant decreases in precipitation. However, there were also regions that experienced increases in precipitation in the range below 4.8 mm, such as Tabora and Katavi. Overall, the results suggest a diverse pattern of variability in precipitation trends across Tanzania over the past five decades.

#### Change and significance trend per decade

The result of the change and significance for maximum (left-middle), minimum (middle) and precipitation (right-middle) (Fig. 4) show that, there has been a significant change in precipitation trends across Tanzania over the decades, with most regions showing a consistent trend of 100%, indicating an increase in precipitation. For maximum and minimum temperatures, the trend per decade significance remained consistent at 100% across all regions, suggesting stable temperature trends. The only exception was Dodoma, Singida and Ruvuma where the trend per decade significance for precipitation was between 95 and 98%, indicating a slightly lower level of significance. Overall, the findings suggest an increase in precipitation trends across Tanzania over the decades, with temperature trends remaining stable.

### Variability and change in variability

#### Temperatures and precipitation

In order to visualize potential shifts in the distribution and variability of climate, this study examines successive climatological periods by comparing changes in the average and dispersion of climate patterns. Each Gaussian distribution curve symbolizes a 30-year climatology span (Fig. 4, bottom), allowing for the identification of trends such as increasing temperatures and the heightened frequency of extreme temperature events.

The variability in minimum temperature (Fig. 4, bottom-middle), shows a slight increase from 1951–1980 to 1991–2020, with the lowest variability in 197–2000. Similarly, the variability in maximum temperature (Fig. 4, bottom-left) also exhibits a slight increase from 1951–1980 to 1991–2020, with the lowest variability in 1971–2000. In contrast, the variability in precipitation remains constant at 0.0 over the three time periods, indicating a consistent and low variability in precipitation levels (Fig. 4, bottom-right). Furthermore, while the variability in temperature data shows a slight increase over the years, the variability in precipitation remains consistently low. This suggests that temperature patterns may be undergoing more noticeable changes compared to precipitation patterns over the time periods studied.

### Climate projections

#### Mean temperatures

**Figure 5:**
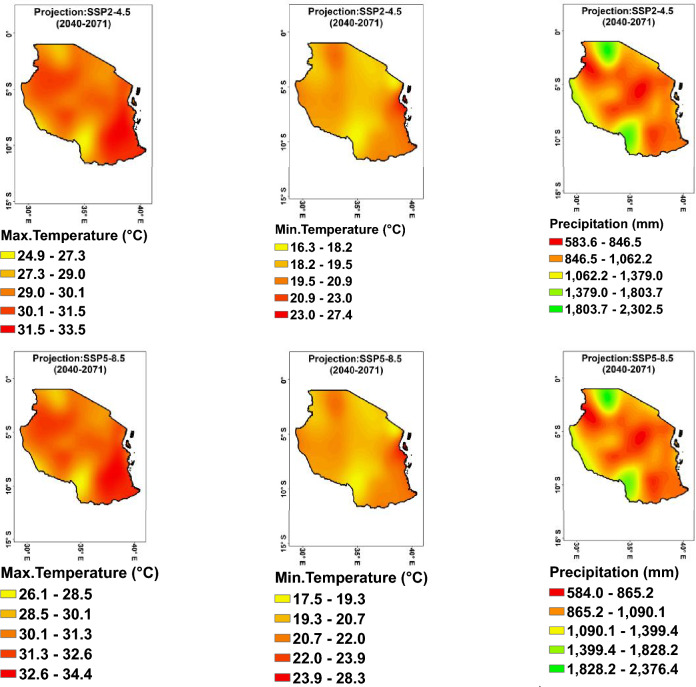
Projected maximum (left), minimum (middle) temperatures, and rainfall (right) variability trend per decade during the period (2040–2071), ensemble mean: SSP2–4.5 (upper) and SSP5–8.5 (lower) scenarios.

The key findings on projected mean minimum, maximum temperatures across Tanzania from 2040 to 2071 under scenarios SSP2–4.5 and SSP5–8.5 (Fig. 5) show that, minimum temperatures across various regions of Tanzania range from 16.3 °C in Njombe to 28.3 °C in Zanzibar. Regions like Lindi and Mtwara are projected to have the highest minimum temperature in both scenarios, at 22.2 °C and 22.6 °C respectively, under SSP2–4.5, and 23.2 °C and 23.6 °C respectively under SSP5–8.5, while regions like Arusha and Njombe are expected to have the lowest minimum temperature (Fig. 5, middle). On the other hand, the projected maximum temperature is expected to increase across Tanzania, with regions like Lindi and Mtwara projected to have the highest maximum temperature of 31.9 °C to 32.9 °C (Fig. 5, left). These findings highlight the potential impacts of climate change on temperature patterns in Tanzania.

The projected temperature increase in Tanzania could have significant impacts on agriculture and water resources in the country. The minimum temperature plays a crucial role in determining the health and growth of crops. When the minimum temperature is too low, it can lead to frost damage on sensitive crops, affecting their yield and quality^79,80^. In regions like Arusha and Njombe with low minimum temperatures, certain crops may struggle to thrive, impacting the overall agricultural productivity in those areas. On the other hand, high minimum temperatures, as seen in Zanzibar, can also have negative effects on agriculture. Elevated minimum temperatures can lead to stress on crops, affecting their growth and development^81^. Some crops may experience heat stress, impacting their ability to produce quality yields^82^. Similarly, the maximum temperature can also influence agricultural activities in Tanzania. High maximum temperatures, such as those projected in Lindi and Mtwara, can increase the risk of heat stress on crops, particularly during hot and dry seasons. Excessive heat can lead to wilting, reduced photosynthesis, and, in severe cases, crop failure. This can affect food security and the livelihoods of farmers who rely on these crops for in come^9^. Conversely, cooler maximum temperatures in certain regions may limit the growth of heat-loving crops or extend the growing season for cool-season crops. Farmers in these areas may need to adapt their crop selection and farming practices to align with the weather conditions to ensure successful harvests. Overall, the projected temperatures in Tanzania can pose challenges to agricultural production^9,80,83,84^.

#### Mean precipitation

The projected precipitation across Tanzania from 2040 to 2071 under SSP2–4.5 and SSP5–8.5 (Fig. 5, right) scenarios is as follows: Under the SSP2–4.5 scenario, projected precipitation values vary across different regions in Tanzania, with the highest values recorded in Njombe (2085.5 mm) and Mwanza (2070.4 mm), and the lowest in Dodoma (628.9 mm).When considering the SSP5–8.5 scenario, the precipitation values also show variation across regions, with Njombe (2035.5 mm) and Mwanza (2132.8 mm) having the highest values, and Dodoma (641.9 mm) having the lowest. Overall, both scenarios show an increase in projected precipitation for most regions in Tanzania, with some areas experiencing more significant increases compared to others. These findings have important implications for water resource management, agriculture, and infrastructure planning in Tanzania in the coming decades.

#### Anomaly change temperatures projection

**Figure 6:**
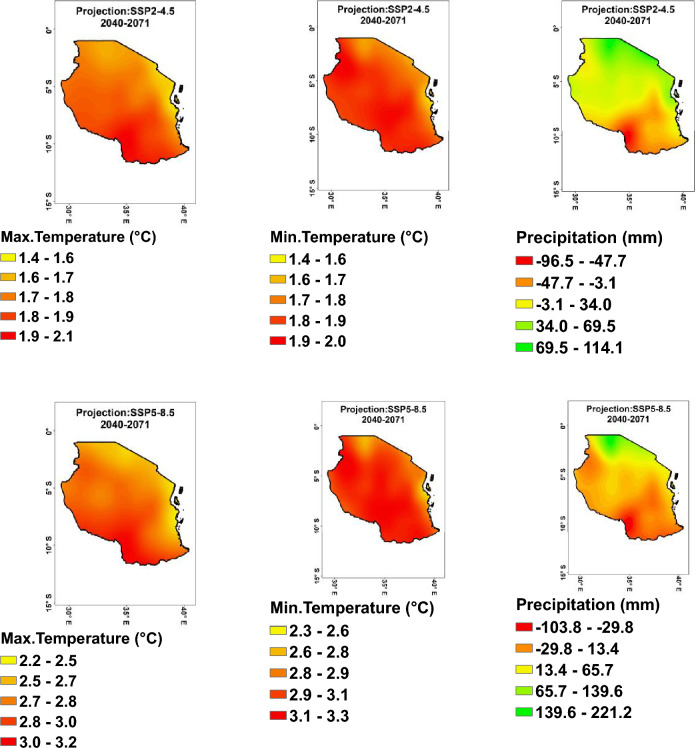
Projected anomaly of maximum (left), minimum (middle) temperatures, and precipitation (right) under SSP2–4.5 (upper) and SSP5–8.5 (lower) scenarios during the period 2040–2071.

The projected anomaly minimum, maximum temperatures in Tanzania from 2040 to 2071 (Fig. 6), indicate that under the SSP2–4.5 scenario, minimum, maximum temperatures are projected to increase, with values ranging from 1.4 to 2.1 °C. Under the more severe SSP5–8.5 scenario, even higher increases in minimum temperature are projected, with values ranging from 2.3 to 3.3 °C. This suggests that Tanzania is likely to experience significant warming if global greenhouse gas emissions continue at the current rate or increase, potentially impacting the environment, agriculture, and human health.

The projected temperature increases in Tanzania under both the SSP2–4.5 and SSP5–8.5 scenarios can be compared to global averages to provide context. The projected temperature increases in Tanzania, ranging from 1.4 to 2.1 °C under the SSP2–4.5 scenario, are expected to be slightly lower than the global average temperature increase. The global average temperature increase under the SSP2–4.5 scenario is projected to be in the range of 1.5 °C to 2.7 °C above pre-industrial levels by 2071. Therefore, Tanzania’s projected temperature increases are generally in line with or slightly lower than the global average under the SSP2–4.5 scenario. Under the SSP5–8.5 scenario, the projected temperature increases in Tanzania, ranging from 2.3 to 3.3 °C, are likely to be lower than the global average temperature increase. The global average temperature increase under the SSP5–8.5 scenario is projected to be in the range of 3.3 °C to 5.7 °C above pre-industrial levels by 2071^1,2,57^. Therefore, Tanzania’s projected temperature increases under the SSP5–8.5 scenario are relatively lower than the global average temperature.

The projected temperature increases in certain regions of Tanzania under the SSP5–8.5 scenario can be attributed to various factors, including: Greenhouse gas emissions. The SSP5–8.5 scenario represents a high emissions pathway where greenhouse gas concentrations, particularly carbon dioxide, are elevated. The continuous release of greenhouse gases into the atmosphere leads to a stronger greenhouse effect, trapping more heat and causing global temperatures to rise. Changes in land use, such as deforestation, urbanization, and agricultural expansion, can contribute to local temperature increases. Deforestation reduces the cooling effect of forests and natural vegetation, leading to higher surface temperatures in the affected areas. Albedo Effect, Changes in surface reflectivity, known as albedo, can influence local temperature patterns. Dark surfaces, such as asphalt and buildings, absorb more solar radiation and generate heat, contributing to higher temperature in urbanized areas. Atmospheric Circulation Patterns: Changes in atmospheric circulation patterns can also influence regional temperature variations. Factors such as air masses, wind patterns, and pressure systems can affect the distribution of heat across different regions, leading to localized temperature changes^1,85,86^. These factors interact in complex ways to contribute to the projected temperature increases in specific regions of Tanzania under the SSP5–8.5 scenario. It is important to consider these interconnected factors when assessing and planning for climate change impacts in the region.

#### Anomaly change precipitation projection

The projected anomaly precipitation across Tanzania for SSP2–4.5 and SSP5–8.5 scenarios (Fig. 6, right), shows significant variation in precipitation levels across different regions. For SSP2–4.5, regions like Geita, Mwanza, and Mara are projected to experience higher precipitation levels in the range of 34.0 mm to 114.1 mm, while regions like Ruvuma, Mtwara, and Mbeya are expected to see a decrease in precipitation in the range of − 3.1 mm to − 96.5 mm. For SSP5–8.5, regions like Mara, Mwanza, and Kagera are projected to experience the highest increase in precipitation in the range of 13.4 mm to 221.2 mm, while regions like Ruvuma, Lindi, and Mtwara are expected to see a decrease in precipitation levels in the range of − 29.8 mm to − 103.8 mm. Overall, these findings highlight the potential impact of different climate scenarios on precipitation patterns in Tanzania. The precipitation anomalies in different regions of Tanzania are influenced by various factors, including topography, proximity to bodies of water, atmospheric circulation patterns, and global climate change^42^.

#### Anomaly changes seasonal projection temperatures

The result indicates that, there is a projected increase in seasonal minimum, maximum temperature anomalies across Tanzania from 2040 to 2071. The minimum temperature anomaly is expected to be higher for the SSP5–8.5 scenario in the range of 2.1 °C to 2.2 °C compared to the SSP2–4.5 scenario for all seasons (Table 4). Similarly, the maximum temperature anomaly is projected to be higher for the SSP5–8.5 scenario in the range of 2.1 °C to 2.5 °C compared to the SSP2–4.5 scenario for all seasons (Table 5). This suggests that Tanzania is likely to experience warmer temperatures in the future, with the SSP5–8.5 scenario leading to greater temperature increases compared to the SSP2–4.5 scenario.

**Table 4:** Projected changes (anomaly) in seasonal minimum temperature under SSP2–4.5 (left) and SSP5–8.5 (right) scenarios.

Regions	Mid-century 2040–2071 change SSP2–4.5 (°C)				Mid-century 2040–2071 change SSP5–8.5 (°C)			
	JF	MAM	JJA	OND	JF	MAM	JJA	OND
Geita	1.8	2.0	2.1	1.9	2.8	3.0	3.2	3.0
Iringa	1.8	1.9	2.1	2.1	2.9	3.1	3.4	3.2
Kagera	1.7	1.8	2.0	1.8	2.6	2.8	3.1	2.9
Katavi	1.7	2.0	2.0	2.0	2.8	3.2	3.3	3.1
Mwanza	1.6	1.7	1.9	1.7	2.5	2.7	3.0	2.7
Njombe	1.8	1.9	2.0	2.0	2.9	3.1	3.2	3.3
Rukwa	1.7	1.9	2.0	2.2	2.8	2.9	3.2	3.2
Shinyanga	1.8	1.9	2.0	1.9	2.9	3.0	3.2	3.0
Simiyu	1.8	1.9	2.0	1.8	2.9	2.9	3.2	2.9
Arusha	1.7	1.8	1.9	1.7	2.7	2.8	3.1	2.8
Dar-es-salaam	1.5	1.6	1.6	1.4	2.4	2.5	2.5	2.3
Dodoma	1.8	1.8	2.1	1.9	2.9	3.0	3.2	3.1
Kigoma	1.7	2.0	2.1	1.9	2.8	3.1	3.2	3.1
Kilimanjaro	1.6	1.7	1.9	1.6	2.5	2.7	2.8	2.6
Lindi	1.7	1.8	1.8	1.7	2.7	2.9	3.0	2.7
Manyara	1.7	1.8	2.0	1.8	2.7	2.8	3.1	2.9
Mara	1.6	1.7	1.9	1.6	2.5	2.7	2.9	2.7
Mbeya	1.7	1.8	2.0	2.1	2.9	3.1	3.2	3.3
Morogoro	1.7	1.8	2.0	1.9	2.8	3.0	3.2	3.0
Mtwara	1.7	1.8	1.8	1.7	2.8	2.8	2.8	2.7
Tanga	1.6	1.6	1.8	1.6	2.6	2.6	2.8	2.6
Ruvuma	1.8	1.9	2.0	2.0	2.9	3.1	3.2	3.1
Singida	1.8	1.9	2.1	2.0	2.9	3.1	3.3	3.2
Zanzibar	1.4	1.5	1.5	1.3	2.3	2.4	2.3	2.1

**Table 5:** Projected changes (anomaly) in seasonal maximum temperature under SSP2–4.5 (left) and SSP5–8.5 (right) scenarios.

Regions	Mid-century 2040–2071 change SSP2–4.5 (°C)				Mid-century 2040–2071 change SSP5–8.5 (°C)			
	JF	MAM	JJA	OND	JF	MAM	JJA	OND
Geita	1.4	1.7	2.0	1.9	2.3	2.7	3.3	2.9
Iringa	1.6	1.8	2.1	2.2	2.5	2.8	3.3	3.3
Kagera	1.4	1.7	2.0	1.7	2.2	2.6	3.1	2.8
Katavi	1.5	1.8	2.1	2.1	2.4	2.7	3.3	3.0
Mwanza	1.4	1.6	2.0	1.7	2.2	2.6	3.1	2.6
Njombe	1.8	1.9	2.0	2.3	2.7	2.9	3.3	3.5
Rukwa	1.6	1.8	2.0	2.2	2.6	2.7	3.3	3.2
Shinyanga	1.4	1.7	2.1	1.8	2.3	2.7	3.3	2.8
Simiyu	1.4	1.6	2.0	1.8	2.3	2.6	3.2	2.6
Arusha	1.4	1.6	1.9	1.8	2.2	2.6	3.0	2.6
Dar-es-salaam	1.5	1.5	1.6	1.5	2.2	2.5	2.5	2.4
Dodoma	1.5	1.7	2.1	2.0	2.4	2.8	3.3	3.1
Kigoma	1.5	1.7	2.0	2.0	2.4	2.6	3.2	3.1
Kilimanjaro	1.3	1.6	1.8	1.7	2.2	2.6	2.7	2.6
Lindi	1.6	1.7	1.9	1.9	2.5	2.7	2.8	2.8
Manyara	1.4	1.7	1.9	1.8	2.3	2.7	3.0	2.7
Mara	1.4	1.6	2.0	1.6	2.2	2.5	3.0	2.5
Mbeya	1.6	1.8	2.1	2.2	2.6	2.9	3.3	3.2
Morogoro	1.6	1.7	2.0	2.0	2.4	2.7	3.1	3.1
Mtwara	1.7	1.7	1.8	1.8	2.7	2.6	2.8	2.8
Tanga	1.3	1.5	1.7	1.7	2.2	2.5	2.7	2.6
Ruvuma	1.8	1.8	1.9	2.2	2.8	2.9	3.2	3.4
Singida	1.5	1.8	2.1	2.0	2.5	2.8	3.3	3.0
Zanzibar	1.4	1.5	1.5	1.3	2.2	2.4	2.3	2.1

The regional variability of temperature anomalies across different parts of Tanzania can be influenced by various factors, such as topography, proximity to water bodies, altitude, and local climatic conditions. Coastal regions such as Dar es Salaam and Zanzibar may experience relatively lower temperature anomalies compared to inland areas due to the moderating effect of the Indian Ocean. However, with the overall warming trend, even coastal regions are expected to see increases in both minimum and maximum temperature anomalies. Northern Highlands: Regions like Arusha and Kilimanjaro, characterized by higher altitudes, may exhibit different temperature anomaly patterns compared to coastal areas. These areas are expected to see an increase in temperature anomalies in the range of 2.2 °C to 3.1 °C. Western and Southern Regions: Areas like Kigoma, Mbeya, and Iringa in the western and southern parts of Tanzania may also experience varying temperature anomalies. These regions can be influenced by factors such as altitude, proximity to Lake Tanganyika, and local weather patterns, leading to regional variability in temperature changes. Central Tanzania: Regions in the central part of the country, including Dodoma and Singida, may show distinct temperature anomaly patterns based on their geographical location and landscape characteristics^87^. The projected temperature anomalies for these areas are in the range of 2.4 °C to 3.3 °C.

#### Anomaly changes seasonal projection precipitation

**Figure 7:**
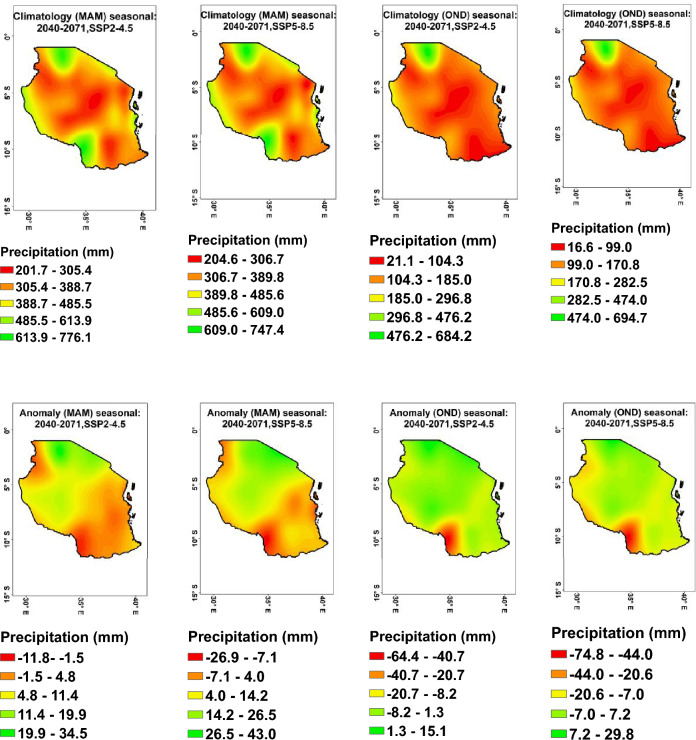
Projected anomaly changes in seasonal precipitation March–April–May (MAM) (bottom-left) and seasonal climatology (top-left); Projected anomaly changes in seasonal precipitation October–November–December (OND) (bottom-left) and its seasonal climatology (top-left) during 2040–2071.

The finding indicates that there will be varying levels of precipitation anomalies across Tanzania from 2040 to 2071 under both the SSP2–4.5 and SSP5–8.5 scenarios (Fig. 7, bottom). The projected precipitation anomalies for different seasons show a general trend of increase or decrease in precipitation levels, with some variability depending on the specific season and scenario. Specifically, the October–November–December (OND) season is expected to see a decrease in precipitation in the range of − 7.0 mm to − 74.8 mm in the southern part of the country and an increase in the range of 1.3 mm to 29.8 mm in the north eastern, and western parts of the country. The March–April–May (MAM) season is expected to see the highest slight decrease in the range of − 1.5 mm to − 26.9 mm in the southern part and the highest increase in the range of 4.8 mm to 43.0 mm under both scenarios in the north eastern.

The projected changes in precipitation in Tanzania may have a significant impact on agriculture in the country. Decreased rainfall could lead to water scarcity and drought, impacting crop production and food security, specifically in southern regions. On the other hand, increased rainfall regions like Mwanza, Kagera, and Mara (northern) could lead to flooding and waterlogging, affecting crop growth and quality. These changes in precipitation patterns could potentially disrupt agricultural activities, reduce crop yields, and threaten the livelihoods of farmers in Tanzania^83^.

#### Projected maximum of daily temperature, by season

The projected maximum daily temperature across Tanzania from 2040 to 2071 (Table 6) shows that, under the SSP2–4.5 scenario, the highest temperature is expected in October–November-December (OND) at 35.5 °C, followed by January–February (JF) at 35.1 °C. For the SSP5–8.5 scenario, the highest temperature is projected in October–November–December at 35.9 °C, followed by January–February at 35.6 °C. Overall, temperatures are expected to increase across all seasons in both scenarios, with higher temperatures projected under the SSP5–8.5 scenario compared to SSP2–4.5.

**Table 6:** Projected maximum daily temperature, by season, from 2040 to 2071.

Scenarios	2040–2071			
Units (°C)	JF	MAM	JJAS	OND
SSP2–4.5	35.1	33.9	32.8	35.5
SSP5–8.5	35.6	34.5	33.5	35.9

### Future trend per decade and change in distribution

#### Projected trend per decade of minimum and maximum temperatures

**Figure 8:**
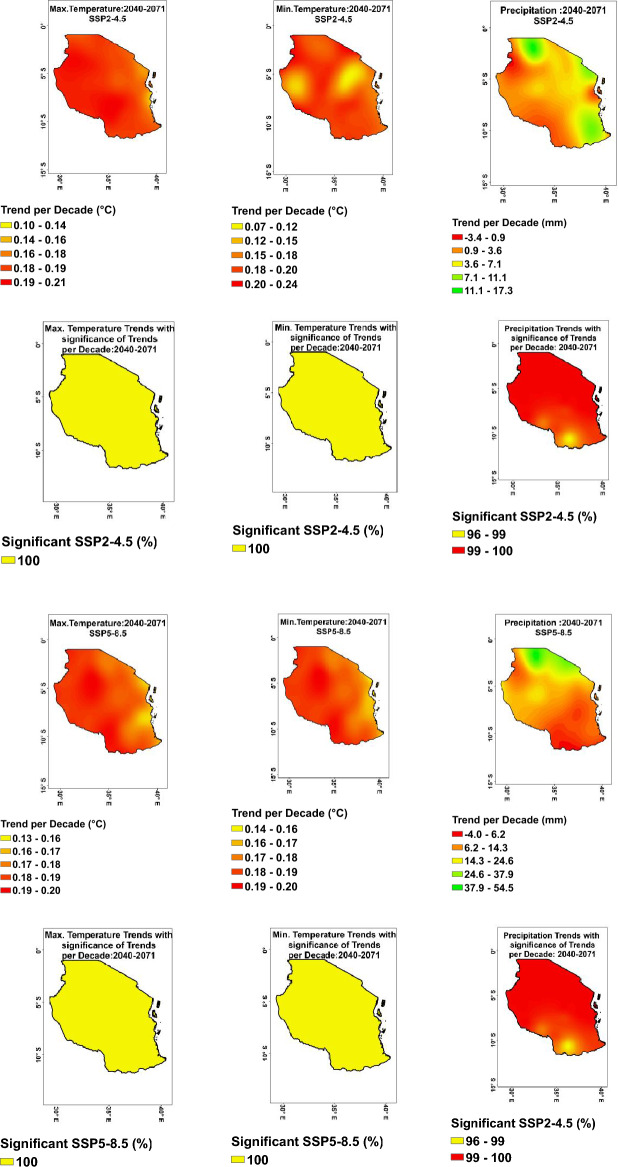
Projected trend per decade and significance of trends per decade for maximum (left), minimum (middle) temperatures, and precipitation (right) under both scenarios SSP2–4.5 (top) and SSP5–8.5 (bottom) during 2040–2071.

The key findings on projected the trend in minimum temperature per decade from 2040 to 2071 under the Shared Socioeconomic Pathways (SSP) 2–4.5 and SSP 5–8.5 include: Under SSP2–4.5, the average temperature increase per decade ranges from 0.1 to 0.2 °C across different regions in Tanzania (Fig. 8, top-middle). Regions with higher temperature projections include Shinyanga, Geita, Kigoma, Tabora, Singida, Kilimanjaro, Ruvuma, and Njombe. Regions with lower temperature projections include Katavi, Manyara, Pwani, and Dodoma. Under SSP5–8.5, the average temperature increases per decade ranges from 0.15 to 0.2 °C in different regions (Fig. 8, bottom-middle). Regions with higher temperature projections include Shinyanga, Tabora, Njombe, and Mwanza. Regions with lower temperature projections include Lindi, Mtwara, Pwani, Dar es Salaam, Zanzibar, and Tanga.

For maximum temperature per decade from 2040 to 2071 under the SSP2–4.5, the average projected maximum temperature increases per decade range from 0.14 to 0.21 °C across different regions in Tanzania (Fig. 8, top-right). Regions with higher temperature projections include Iringa, Njombe, Geita, Kigoma, Tabora, Katavi, Rukwa, Mbeya, Shinyanga, and Mara. Regions with lower temperature projections include Dar es Salaam, Zanzibar, Lindi, and Tanga. Under SSP5–8.5, the average projected maximum temperature increases per decade range from 0.15 to 0.20 °C across different regions (Fig. 8, bottom-right). Regions with higher temperature projections include Iringa, Njombe, Geita, Kigoma, Tabora, Katavi, Rukwa, Mbeya, Shinyanga, and Mara. Regions with lower temperature projections include Lindi, Mtwara, Pwani, Dar es Salaam, and Zanzibar.

These findings provide valuable insights into the potential temperature changes that different regions in Tanzania may experience over the coming decades under different socioeconomic pathways.

#### Projected trend per decade of precipitation

Analysing the projected trends for precipitation per decade from 2040 to 2071 under the Shared Socioeconomic Pathways (SSP) 2–4.5 and SSP 5–8.5. Under SSP2–4.5: The average projected precipitation changes per decade vary across regions, with some experiencing increases and others showing decreases. Regions with significant increase projections include Mwanza (15.5 mm), Kilimanjaro (9.87 mm), Geita (7.18 mm), and Kagera (9.44 mm). Regions like Dodoma (5.48 mm), Morogoro (5.09 mm), and Lindi (9.13 mm) also show notable increases. On the contrary, regions like Mbeya (− 0.62 mm), Njombe (− 0.35 mm), and Ruvuma (1.57 mm) show some decreases in precipitation. Under SSP5–8.5: The average projected precipitation changes per decade show more widespread increases across regions compared to SSP2–4.5. Regions with substantial increases in precipitation include Mwanza (47.37 mm), Mara (45.25 mm), Kilimanjaro (37.51 mm), and Arusha (34.49 mm). Regions like Geita (25.56 mm), Kagera (31.54 mm), Kigoma (20.36 mm), and Tanga (15.40 mm) also exhibit significant increases. However, regions like Ruvuma (− 1.47 mm) and Njombe (1.05 mm) still depict decreases in precipitation under this scenario.

#### Significant trend per decade from 2040 to 2071

The key findings on the trends per decade across Tanzania during 2040–2071 for precipitation (Fig. 8-right), show that most regions experienced a significant increase in precipitation, with values ranging from 97 to 100%. For maximum (Fig. 8-left) and minimum (Fig. 8-middle) temperatures during the same period, all regions showed a significant increase in both parameters, with values of 100% for most regions. This indicates a consistent warming trend across Tanzania during the period analysed.

#### Temperatures change within variability

**Figure 9:**
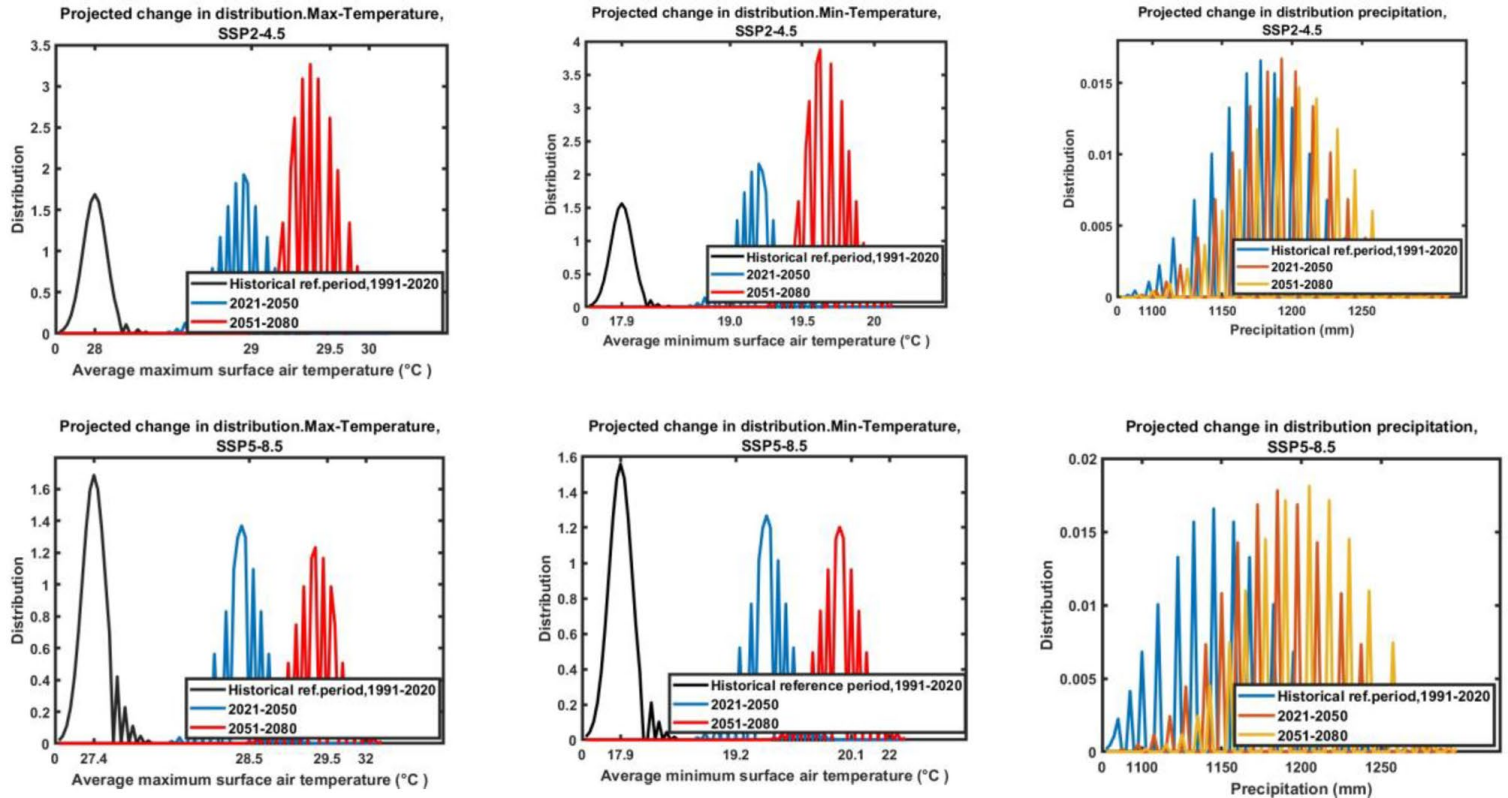
Projected change in distribution average maximum (left), minimum (middle) temperature and precipitation (right) under both scenarios SSP2–4.5 (top) and SSP5–8.5 (bottom) during 2040–2071.

Under the SSP2–4.5 scenario, there is a projected increase in minimum temperature across Tanzania from 2040 to 2071, with a corresponding increase in temperature distribution. On the other hand, under the SSP5–8.5 scenario, there is a smaller projected increase in minimum temperature and a decrease in temperature distribution over the same time period (Fig. 9, middle). In terms of maximum temperature, the analysis shows a similar trend, with increasing maximum temperature and a higher temperature distribution under the SSP2–4.5 scenario, while under the SSP5–8.5 scenario, there is a smaller increase in maximum temperature and a decrease in temperature distribution (Fig. 9, left). Overall, the findings suggest that under both scenarios, Tanzania is likely to experience an increase in temperatures, but the extent of the increase and distribution will vary depending on the scenario.

The distribution of minimum, maximum temperatures in Tanzania, with distribution levels consistently at 1.5 °C to 3.5 °C under both scenarios (Fig. 9), is significant for several reasons: Agricultural Impact, the distribution of temperatures directly affects agriculture in Tanzania. Crops have specific temperature requirements for growth and development. A shift in temperature distribution can impact crop yields, planting seasons, and overall agricultural productivity. Temperature distribution influences the availability of water resources in Tanzania. Changes in temperatures can affect precipitation patterns, evaporation rates, and water availability, which are crucial for agriculture, ecosystems, and human consumption^83^. Temperature distribution plays a vital role in shaping ecosystems and biodiversity. Fluctuations in temperature can impact the habitat range of different species, leading to changes in biodiversity and ecosystem functions^88^. Extreme temperatures can have implications for human health in Tanzania. Heatwaves or cold spells resulting from temperatures distribution changes can affect vulnerable populations, leading to health issues such as heatstroke, dehydration, or respiratory problems^19^. Furthermore, understanding the distribution of temperatures in Tanzania is essential for assessing the impacts of climate change. Changes in temperature patterns can provide insights into the extent of climate change effects on the region, enabling policymakers to develop adaptation and mitigation strategies.

#### Precipitation changes within variability

Under the SSP2–4.5 scenario, there is a slight increase in distribution from the historical reference to the future periods (2040–2071), with distribution levels fluctuating between 0.01 and 0.02 (Fig. 9, top-right). On the other hand, under the SSP5–8.5 scenario, there is a more significant increase in distribution precipitation, with distribution levels consistently at 0.02 across all future periods (Fig. 9, bottom-right). Furthermore, the findings suggest that distribution precipitation is projected to increase under both scenarios, with the SSP5–8.5 scenario showing a stronger trend towards improved distribution readiness.

### Changes and significance of temperatures and precipitation from natural variability

**Figure 10:**
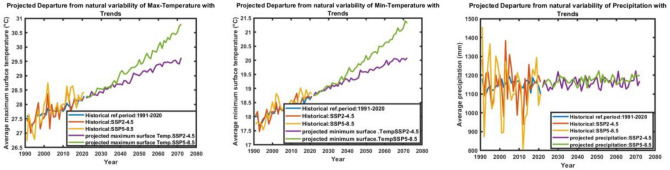
Projected significant change from natural variability for maximum (left), minimum (middle), and precipitation (right) for the year 2040–2071 relative to the historical reference period 1991–2020.

The changes and significance of temperatures and precipitation from natural variability (Fig. 10), indicate a projected increase in minimum temperature for the years 2040–2071 (Fig. 10-middle), with the SSP-5–8.5 scenario predicting higher temperatures compared to the SSP-2–4.5 scenario. There is a slightly lower range of minimum temperature values for SSP-2–4.5 (19.5 °C to 20.9 °C) compared to SSP-5–8.5 (20.5 °C to 21.4 °C). Additionally, the result shows that under the SSP-5–8.5 scenario, there is a wider range of potential maximum temperatures (29.2 °C to 30.5 °C) compared to SSP-2–4.5 (28.5 °C to 29.3 °C) for the year 2040–2071 (Fig. 10-left). This indicates that higher emission scenarios lead to a wider range of maximum temperature predictions. Furthermore, the combination of increased greenhouse gas emissions, land use changes, industrial activities, feedback mechanisms, and atmospheric circulation patterns in the SSP-5–8.5 scenario collectively contributes to the higher temperatures predicted for the future.

Additionally, the result indicates that for the scenario SSP-2–4.5, the ensemble range of precipitation for the 10-90th percentile in the year 2040–2071 is between 1106.1 and 1150.8 mm. In comparison, for the scenario SSP-5–8.5, the ensemble range of precipitation for the same percentile range and year is between 1100.7 and 1160.7 mm (Fig. 10-right). This suggests that there is a slight decrease in the lower bound of the precipitation range under the higher emissions scenario SSP-5–8.5 compared to the lower emissions scenario SSP-2–4.5. This change in the range of precipitation may have significance in understanding the potential impacts of different climate scenarios on regional water resources and ecosystems.

## Conclusion

Our study provides valuable insights into future climate projections across Tanzania, emphasizing the need for proactive measures to address the potential impacts of climate change. The observed changes in temperatures and precipitation patterns underscore the urgency of implementing adaptation strategies to enhance the resilience of vulnerable sectors such as agriculture and water resources.

The key findings of the research on future climate projections across Tanzania using CMIP6 and a high-resolution regional climate model (ICTP-RegCM4-3, MPI-CSC-REMO2009, and CCCma-CanRCM4) showed that there is a significant gap in previous research in terms of predicting future climate trends at a regional level. This study used more advanced models to provide more detailed and reliable projections, which help inform climate change adaptation strategies in Tanzania. Based on the research findings regarding future climate trend per decade projections in Tanzania, Regions with higher temperature projections include Iringa, Njombe, Geita, Kigoma, Tabora, Katavi, Rukwa, Mbeya, Shinyanga, and Mara. Regions with lower temperature projections include Dar es Salaam, Zanzibar, Lindi, and Tanga under both scenarios. Furthermore, Under SSP2–4.5: The average projected precipitation changes per decade vary across regions, with some experiencing increases and others showing decreases. Regions with significant increase projections include Mwanza (15.5 mm), Kilimanjaro (9.87 mm), Geita (7.18 mm), and Kagera (9.44 mm). Regions like Dodoma (5.48 mm), Morogoro (5.09 mm), and Lindi (9.13 mm) also show notable increases. On the contrary, regions like Mbeya (− 0.62 mm), Njombe (− 0.35 mm), and Ruvuma (1.57 mm) show some decreases in precipitation. Under SSP5–8.5: The average projected precipitation changes per decade show more widespread increases across regions compared to SSP2–4.5. Regions with substantial increases in precipitation include Mwanza (47.37 mm), Mara (45.25 mm), Kilimanjaro (37.51 mm), and Arusha (34.49 mm). Regions like Geita (25.56 mm), Kagera (31.54 mm), Kigoma (20.36 mm), and Tanga (15.40 mm) also exhibit significant increases. However, regions like Ruvuma (− 1.47 mm) and Njombe (1.05 mm) still depict decreases in precipitation under this scenario.

Certain adaptation strategies have the potential to reduce the negative effects of climate change: Water Management Strategies. Implementing water management strategies such as rainwater harvesting, improved irrigation techniques, and water conservation practices can help communities cope with changes in precipitation patterns and mitigate the effects of droughts and water scarcity. Crop diversification, encouraging farmers to diversify their crops and adopt climate-resilient varieties can help ensure food security in the face of changing climate conditions. This can include promoting drought-resistant crops and sustainable agricultural practices. Forestry and Land Management, implementing reforestation and afforestation programmes, as well as sustainable land management practices, can help mitigate the impacts of deforestation and land degradation, while also contributing to carbon sequestration and biodiversity conservation. Infrastructure Resilience. Building climate-resilient infrastructure, such as flood barriers, improved drainage systems, and resilient buildings, can help reduce the vulnerability of communities to extreme weather events like floods and storms. Community-Based Adaptation. Engaging local communities in participatory adaptation planning processes can help identify context-specific adaptation options that are tailored to the needs and priorities of the communities most at risk. Early Warning Systems, strengthening early warning systems for extreme weather events can help communities prepare and respond effectively to disasters, reducing the risks to lives and livelihoods. Capacity Building and Education, investing in capacity building, training, and education programmes on climate change adaptation can empower communities and local authorities to take proactive measures to reduce their vulnerability to climate risks. Ecosystem-Based Adaptation, Protecting and restoring natural ecosystems such as forests, wetlands, and coastal habitats can provide multiple benefits, including flood regulation, carbon sequestration, and biodiversity conservation, while enhancing the resilience of communities to climate change impacts. Cross-Sectoral Collaboration. Promoting collaboration and coordination among different sectors, stakeholders, and levels of government can help ensure integrated and holistic approaches to climate change adaptation that address the diverse challenges faced by Tanzania’s communities. Additionally, policymakers and stakeholders should prioritize sustainable development practices that promote climate resilience and mitigate the adverse effects of climate change on Tanzania’s economy and society. By integrating these findings into decision-making processes, Tanzania can better prepare for future climate challenges and work towards a more sustainable and climate-resilient future.

### Supplementary Information

Supplementary Table S1.

## Data Availability

The data generated during analysis are available via request from email: 202251080003@nuist.edu.cn.
