# Supplementary material for: Future climate projection across Tanzania under CMIP6 with high-resolution regional climate model
Source: Sci Rep. 2024 Jun 3;14:12741. doi: 10.1038/s41598-024-63495-w (PMC11148196; doi:10.1038/s41598-024-63495-w)
Supplement: Supplementary file 1 — Supplementary Table S1. [file 41598_2024_63495_MOESM1_ESM.docx]

| **Table S1**. Information about the selected representative regions (Meteorological stations). | | | | |
| --- | --- | --- | --- | --- |
| **No.** | **Station Name** | **Latitude**  **(°)** | **Longitude**  **(°)** | **Altitude**  **(m)** |
| 1 | Geita | -2.8 | 32.2 | 1240 |
| 2 | Iringa | -7.7 | 35.7 | 1428 |
| 3 | Kagera | -1.3 | 31.8 | 1144 |
| 4 | Katavi | -6.3 | 31.2 | 1502 |
| 5 | Mwanza | -2.5 | 32.9 | 1140 |
| 6 | Njombe | -9.3 | 34.8 | 1821 |
| 7 | Rukwa | -8.0 | 31.4 | 800 |
| 8 | Shinyanga | -3.6 | 33.4 | 1127 |
| 9 | Simiyu | -2.8 | 34.1 | 1377 |
| 10 | Arusha | -3.4 | 36.6 | 1387 |
| 11 | Dar-es-salaam | -6.8 | 39.2 | 53 |
| 12 | Dodoma | -6.2 | 35.8 | 1120 |
| 13 | Kigoma | -4.9 | 29.6 | 820 |
| 14 | Kilimanjaro | -3.0 | 37.3 | 813 |
| 15 | Lindi | -10.0 | 39.7 | 402 |
| 16 | Manyara | -4.3 | 36.9 | 1197 |
| 17 | Mara | -1.5 | 33.8 | 1147 |
| 18 | Mbeya | -8.9 | 33.4 | 1758 |
| 19 | Morogoro | -6.8 | 37.6 | 526 |
| 20 | Mtwara | -10.2 | 40.1 | 113 |
| 21 | Tanga | -5.0 | 39.0 | 49 |
| 22 | Ruvuma | -10.6 | 36.2 | 1036 |
| 23 | Singida | -4.8 | 34.7 | 1260 |
| 24 | Zanzibar | -6.2 | 39.2 | 18 |
